# Supplementary material for: VIOLIN: A modular framework for scalable reconciliation of heterogeneous interaction graphs
Source: bioRxiv. 2026 Mar 28:2024.07.21.604448. Preprint. [Version 3] doi: 10.1101/2024.07.21.604448 (PMC13041817; doi:10.1101/2024.07.21.604448)
Supplement: Supplement 1 [file media-1.pdf]

Supplementary Material

1 Formal definitions and notation

This section provides the formal definitions underlying VIOLIN's reconciliation algorithm.

1.1 Element, interaction, and path definitions

Several definitions in this sub-section are adopted from the BioRECIPE documentation[1,2] and are included here to provide notation context for new definitions. Table S1 includes five examples of element and interaction attribute values. More examples can also be found in GitHub repositories for BioRECIPE[3] and VIOLIN[4].

Definition 1.1. An ***element*** $v = v(\mathbf{a}^v)$, is defined by its name (a^{name}), type (a^{type}), and a unique database identifier (ID) ($a^{database}$ and a^{ID}) or the gene symbol from the HGNC database ($a^{HGNCsymbol}$). These five attributes are referred to as *essential element attributes*. Other element attributes are the element subtype $a^{subtype}$, the cellular compartment $a^{compartment}$ and the compartment's unique ID $a^{compartmentID}$. Element attributes are written as a vector:

$$\mathbf{a}^v = \left(a^{name}, a^{type}, a^{subtype}, a^{HGNCsymbol}, a^{database}, a^{ID}, a^{compartment}, a^{compartmentID}\right).$$

Definition 1.2. A ***directed signed interaction*** $e = e(v_s, v_t, \mathbf{a}^e)$ is defined with its source element v_s, target element v_t, and a vector of attributes $\mathbf{a}^e$. The *essential interaction attribute* is the interaction sign a^{sign}. The direction of an interaction is always implicitly defined with its source and target nodes, and therefore, not explicitly listed among its attributes. Other interaction attributes that further define the **influence** are the interaction connection type $a^{connectiontype}$ indicating whether the influence is direct or indirect, the mechanism of the interaction $a^{mechanism}$, and the molecular site of the interaction a^{site}. The **context** attributes include cell line $a^{cellline}$, cell type $a^{celltype}$, tissue type $a^{tissuetype}$, and organism $a^{organism}$ where the interaction occurs. The **provenance** attributes include the belief or confidence score for the interaction a^{score}, the source from which the interaction is obtained a^{source}, the statements $a^{statements}$ and IDs of papers $a^{paperIDs}$ where the interaction is mentioned. These attributes together form the interaction attribute vector:

$$\mathbf{a}^{\mathrm{e}} = (a^{sign}, a^{connectiontype}, a^{mechanism}, a^{site}, a^{cellline}, a^{celltype}, a^{tissuetype}, a^{organism}, a^{score}, a^{source}, a^{statements}, a^{paperIDs}).$$

Definition 1.3. A ***path*** in a model is formed from $n > 1$ connected edges: $p\left(v_{s_p}, v_{t_p}, a^{sign_p}\right) = (e(v_{s_p} = v_{k_1}, v_{k_2}, \mathbf{a}^e_{k_1}),\ e(v_{k_2}, v_{k_3}, \mathbf{a}^e_{k_2}), \dots, e(v_{k_n}, v_{k_{n+1}} = v_{t_p}, \mathbf{a}^e_{k_n}))$. The direction of the path is

implicitly defined with the source node v_{s_p} and target node v_{t_p}. The regulation sign a^{sign_p} is considered positive when the number of negative signs in the set $\{a_{k_1}^{sign}, a_{k_2}^{sign}, .., a_{k_n}^{sign}\}$ is even, and negative when this number is odd.

Definition 1.4. A directed signed interaction $e = e(v_s, v_t, \mathbf{a}^e)$ where $v_s \equiv v_t$ is called ***direct self-regulation***.

Definition 1.5. A path $p(v_{s_p}, v_{s_p}, a^{sign_p})$ where the path source is also the path target, $v_{s_p} \equiv v_{s_p}$, is called a ***feedback loop***. If a^{sign_p}="positive", it is a positive feedback loop and if a^{sign_p}="negative", it is a negative feedback loop.

1.2 Match definitions

Definition 2.1. Two elements $v = v(\mathbf{a}^v)$ and $v' = v'(\mathbf{a}^{v'})$ satisfy the ***necessary element match condition*** if they have at least one of the following, the same HGNC symbol ($a^{HGNCsymbol} = a^{HGNCsymbol'}$) or the same name ($a^{name} = a^{name'}$) or the same unique database ID ($a^{database} = a^{database'}$ and $a^{ID} = a^{ID'}$), and they also have the same element type ($a^{type} = a^{type'}$).

Definition 2.2. Two interactions, $e(v_s, v_t, \mathbf{a}^e)$ and $e'(v'_s, v'_t, \mathbf{a}^{e'})$, satisfy the ***necessary interaction match condition*** if elements v_s and v_t satisfy the necessary condition to match elements v'_s and v'_t, respectively, and the interaction signs are same ($a^{sign} = a^{sign'}$).

Definition 2.3. If the corresponding attributes in two interactions are different (e.g., $a^{location} \neq a^{location'}$), we refer to this as ***attribute mismatch***. We note here that, in the case when the attribute value in one of the two interactions is "empty", this will not be considered as an attribute mismatch.

Definition 2.4. An interaction $e'(v'_s, v'_t, \mathbf{a}^{e'})$ satisfies the ***necessary path match condition*** to match a path in a model, if $a^{connectiontype'}$ ="indirect", there exists a path $p\left(v_{s_p}, v_{t_p}, a^{sign_p}\right)$ in the model, nodes v'_s and v'_t satisfy the necessary conditions to match two nodes v_{s_p} and v_{t_p}, and the sign of the interaction matches the sign of the path $a^{sign'} = a^{sign_p}$.

1.3 Corroboration definitions

The definitions in sections 1.3-1.6 assume the default classification scheme CS1, and classification attribute choice strategy CA1. When additional conditions are included for other schemes (CS2, CS3) or strategies (CA2, CA3, CA4), a NOTE following the definition describes these differences.

Definition 3.1. An interaction $e'(v'_s, v'_t, \mathbf{a}^{e'})$ is classified as ***weak corroboration*** of a model interaction $e(v_s, v_t, \mathbf{a}^e)$, if e and e' satisfy the necessary interaction match condition and one of the following conditions is satisfied:

i) ***empty attribute***: there is no mismatch between corresponding attributes in $\mathbf{a}^e$ and $\mathbf{a}^{e'}$, and at least one of the non-essential attributes in $\mathbf{a}^{e'}$ is "empty".

ii) ***indirect interaction***: there is no mismatch between corresponding attributes in $\mathbf{a}^e$ and $\mathbf{a}^{e'}$, except e' is an indirect interaction, $a^{connectiontype'}$ = "indirect" and e is a direct interaction, $a^{connectiontype}$="direct".

Definition 3.2. An interaction $e'(v'_s, v'_t, \mathbf{a}^{e'})$ with $a^{connectiontype'}$ = "indirect" is classified as ***path corroboration*** if there is no such interaction $e(v_s, v_t, \mathbf{a}^e)$ in the model that satisfies necessary element match conditions with e', and instead e' satisfies the necessary condition to match a path $p\left(v_{s_p}, v_{t_p}, a^{sign_p}\right)$ in the model.

Definition 3.3. An interaction $e'(v'_s, v'_t, \mathbf{a}^{e'})$ is classified as ***strong corroboration*** of a model interaction $e(v_s, v_t, \mathbf{a}^e)$, if e and e' satisfy the necessary interaction match condition, $a^{connectiontype'}$ = $a^{connectiontype}$ and the $a^{compartment'}$ and $a^{compartmentID'}$ attribute values of both v'_s and v'_t match the values of corresponding attributes in v_s and v_t, respectively (NOTE: in the other three classification strategies, additional attributes are included in the condition, specifically, the mechanism attribute for CA2, mechanism and cell line attributes for CA3, and mechanism and all context attributes for CA4).

Definition 3.4. An interaction $e'(v'_s, v'_t, \mathbf{a}^{e'})$ is classified as ***specification*** if there exists a model interaction $e(v_s, v_t, \mathbf{a}^e)$, such that e and e' satisfy the necessary interaction match condition, and either

i) all remaining attributes match such that there is at least one "empty" model interaction attribute and a non-"empty" corresponding interaction attribute, or

ii) $a^{connectiontype'}$="direct" and $a^{connectiontype}$="indirect" and all the remaining attributes match.

1.4 Contradiction definitions

Definition 4.1. An interaction $e'(v'_s, v'_t, \mathbf{a}^{e'})$ is classified as ***contradiction*** if there exists a baseline model interaction $e(v_s, v_t, \mathbf{a}^e)$ such that:

i) ***direction contradiction***: v'_t satisfies the necessary condition to match v_s, v'_s satisfies the necessary condition to match v_t, and either the model interaction is indirect

($a^{connectiontype}$=”indirect”), or the model interaction is direct ($a^{connectiontype}$=”direct”) and there is a mismatch between e' and e in at least one non-essential attribute. (NOTE: in the CS3 scheme, this is considered a flagged interaction, if there is also a sign mismatch, Figure 3).

ii) ***sign contradiction***: v'_s satisfies the necessary condition to match v_s, v'_t satisfies the necessary condition to match v_t, and there is a mismatch in signs between e and e', that is, $a^{sign} \neq a^{sign'}$. (NOTE: in the CS3 scheme, when $a^{connectiontype'}$ =”indirect” and $a^{connectiontype}$=”direct” this is considered a flagged interaction, Figure 3).

iii) ***attribute conflict***: e and e' satisfy the necessary match conditions, and there is a mismatch between e and e' in at least one non-essential attribute other than $a^{connectiontype'}$.

The reasoning behind the connection type requirement in the direction contradiction definition is that an indirect model interaction may be a “placeholder” in the absence of more detailed knowledge, and the new interaction can dispute this previously incomplete knowledge. On the other hand, when the model interaction is direct, and there is a contradiction with the new interaction in direction and other attributes (e.g., location), this can indicate a potential error in the interaction extraction tool or an incorrect model interaction.

1.5 Extension definitions

Definition 5.1. An interaction $e'(v'_s, v'_t, \mathbf{a}^{e'})$ is classified as ***extension*** in the following cases:

iii) ***internal extension***: if there exist two different model elements v_i and v_j such that v'_s and v'_t satisfy at least the necessary condition to match v_i and v_j, respectively, there is no interaction $e(v_i, v_j)$ or interaction $e(v_j, v_i)$ in the model, and either $a^{connectiontype'}$ =”direct”, or $a^{connectiontype'}$=”indirect” and there is no path $p(v_i, v_j)$ in the model. (NOTE: in the CS2 scheme, when $a^{connectiontype'}$=”direct”, this is considered a flagged interaction, Figure 3).

iv) ***hanging extension***: if either v'_s or v'_t (and not both) matches a model element.

v) ***full extension***: there are no baseline model elements that match v'_s and v'_t.

1.6 Flagged definitions

There are some cases where a combination of matched, mismatched, and “empty” attributes requires further manual inspection before it can be classified, and therefore, these interactions are flagged by VIOLIN.

Definition 6.1. An interaction $e'(v'_s, v'_t, \mathbf{a}^{e'})$ is classified as ***flagged*** in the following cases:

i) ***direction mismatch***: if there exists a model interaction $e(v_s, v_t, \mathbf{a}^e)$ with $a^{connectiontype}$ =”direct”, v'_s and v'_t satisfy the necessary condition to match v_t and v_s, respectively, and there is no mismatch in other element attributes and no mismatch in context attributes (“empty” attributes are allowed). (NOTE: in the CS3 scheme, this is in some instances considered a direction contradiction, Figure 3).

ii) ***path mismatch***: if $a^{connectiontype'}$ =”indirect”, nodes v'_s and v'_t satisfy the necessary condition to match two nodes in the baseline model, v_i and v_j, respectively, there is no interaction $e(v_i, v_j)$ or interaction $e(v_j, v_i)$ in the model, and instead there is either a path $p(v_i, v_j, a^{sign_p})$ where $a^{sign'} \neq a^{sign_p}$ or a mismatch in any of the non-essential attributes of the source and target elements, or there is a path $p(v_j, v_i, a^{sign_p})$. (NOTE: in the CS2 scheme, these are considered contradictions, Figure 3).

iii) ***self-regulation***: if e' is a self-regulation ($v_s \equiv v_t$) and there exists a model element v_i such that v'_s and v'_t satisfy the necessary condition to match v_i.

The self-regulation flagged interactions may indeed be self-regulations, but more often the self-regulation is a result of grounding in the interaction extraction tool, and the level of detail and abstraction in the baseline model, and therefore, we flag them. Grounding is an extraction step that uses public databases [5,6] to pair a standard identifier with the common name used in the literature text, though it can occasionally result in error. For example, in the literature, Caspase-3 and Caspase-8 interact with each other as separate entities, while in the melanoma model that we use as one of our case studies, all the caspases are grouped in a single variable. Thus, the interaction between Caspase-3 and Caspase-8 is classified as self-regulation, and the user can then decide how to use the interaction. Self-regulation interactions may also result from the qualifier such as “mutated” or “phosphorylated” being ignored by the reader. For example, the statement “R2834H DP decreases phosphorylation of DP compared with WT” means that the mutated protein is not phosphorylated and therefore decreases the phosphorylation compared to wild type (WT), the extracted event is “DP negatively regulates DP,” ignoring the important distinction of the mutated form.

2 Corpus construction and query design

To generate heterogeneous interaction inputs for reconciliation analysis, we constructed nine literature corpora. These corpora were designed to vary in size, query specificity, contextual alignment with baseline graphs and retrieval strategy with varying degrees of topical alignment to the baseline graphs.

Corpora were assembled using PubMed Central[7] and REACH Explorer[8] queries designed to produce: 1- broad, context-aligned literature set (S_{A1}); 2- protein-centered retrieval strategy (S_{A2}); 3- targeted pathway-

focused literature sets for baseline graphs A and B (S_{A3}, S_{A4}, S_{B1} - S_{B3}); 4- intentionally weakly related ("negative") literature sets (S_{B*1}, S_{B*2}). Query details are provided in Table S2. Relevant corpora were designed to align with the biological contexts represented by the baseline graphs. Negative corpora included terms unrelated to one baseline graph to evaluate reconciliation robustness under irrelevant input conditions. This variation allowed evaluation of VIOLIN under diverse input conditions.

3 Reader extraction behavior

The steps that we used for generating interaction lists are outlined in Figure S3A and the features of the four tools relevant to interaction extraction are summarized in Figure S3B. The prompt used with LLMs is included in Figure S3C. The summary of paper corpus sizes and corresponding interactions list sizes are provided in Table S2. An average runtime per paper across all interaction lists (Figure S3B) indicates that INDRA outperforms the other tools, while GPT-4.1 is the slowest. This difference in speed is expected, given that INDRA draws its output in part from existing databases while GPT-4.1 is based on a very large model. Further, for the largest paper corpus, S_{A1}, we limited analysis to REACH and INDRA only, as applying LLM-based readers to this corpus was computationally impractical due to extended runtimes.

4 Baseline knowledge graphs - context and source

Baseline graph A (melanoma signaling network) was created based on biological pathways in the melanoma SkMel-133 cell line from Korkut et al [9], where the authors performed DNA, RNA, and protein analysis on primary and metastatic melanomas from 331 patients. Their investigation focused on four specific genomic subtypes: mutant BRAF, mutant RAS, mutant NF1, and triple wild type. The goal of both the wet lab experiments and the model analysis was to investigate potential immunotherapies. Additional subtypes exist that may benefit from MAPK and RTK inhibition therapies[9]. Graph topology is relatively dense compared to graph B, with multiple convergent signaling cascades and cross-pathway interactions (Figure S4A).

Baseline graph B (naïve T cell differentiation network) was created based on the published network[10] that captures the circuitry that controls the differentiation of naïve T cells into regulatory (Treg) and helper (Th) cells. Previously published model analysis[10] focused on the early steps of T cell activation, and the suppression or generation of regulatory T cells (Treg), connecting the Akt-mTOR pathway to Treg development and the T cell receptor (TCR) pathways to activation and inhibition of Foxp3, leading to different T cell fates. In contrast to the melanoma SkMel-133 cell network, the T cell network includes almost exclusively protein-protein interactions, with very few chemical and gene nodes. Compared to graph A, this network is smaller and topologically simpler, providing a contrasting structural environment for reconciliation (Figure S4B).

We also classified interaction list R_{A2} with respect to several other models. The summaries of all baseline graphs used for testing VIOLIN are provided in Figure S4C. Results for these additional models are available on GitHub.

5 Detailed classification subcategory distribution

We investigated classification subtype distributions across all readers and all corpora. The distributions and total numbers for each reader in each category are shown in Figure S5.

6 Attribute coverage by readers

In addition to attribute coverage for each reader across all corpora (Figure 3B), we also summarized attribute coverage using two heatmaps (Figure S6). The first heatmap (Figure S6A) shows relative attribute coverage within each extracted interaction list, for each reader and each corpus. The second heatmap (Figure S6B) shows counts of interactions that have non-empty or specific values for mechanism and connection type attributes, colored relative to the highest count for each corpus.

Although finding new direct interactions and including the information about their mechanism is critical for mechanistic modeling, the results suggest that traditional machine readers struggle to determine whether an interaction is direct, and therefore, default to assigning "indirect" to the connection type attribute, while LLM tools are more successful in this task. On the other hand, INDRA assigns values to the mechanism attribute in all extracted interactions. However, given the small number of interactions INDRA finds, Llama 3 extracts the largest number of interactions with Mechanism attribute value in most corpora.

7 Sensitivity to attributes within classification criteria

The classification results highlight the importance of carefully selecting the most relevant literature for modeling and offer insights into the most useful approach for information extraction based on the classification category of interest.

Non-essential attributes may skew the classification outcome. For instance, the baseline graph for SkMel133 cell line includes interaction 'LKB1' activates 'AMP-activated protein kinase (AMPK)' and an extracted interaction states that 'AMPK' is positively regulated by 'LKB1', with the literature evidence linking it to NCI-H460, a Non-Small Cell Lung Cancer (NSCLC) cell line. When only necessary match attributes and the compartment attribute are used (CA1 strategy), the extracted interaction is classified as strong corroboration. When the cell line attribute is included in classification criteria (CA3), this interaction is classified as contradiction due to the conflicting cell line attribute.

When changing from CA1 to CA4, corroborations are reclassified to contradictions and flagged for REACH (57% of corroborations; 1.4% of all interactions) and Llama 3 (40% of corroborations, 2.9% of all interactions), and few being reclassified to internal extensions (Figure 6B). While adding Mechanism to classification strategy in CA2 had very little influence on classification outcomes overall, it was the only change in criteria that affected INDRA output (6 interactions total), as it didn't further change in CA3 and CA4. This is expected, as INDRA does not provide values for contextual attributes such as cell line, cell type, tissue type, or organism (Figure 3B). Interestingly, GPT-4.1 output is not affected by the inclusion of the mechanism attribute in the classification criteria and instead shows its greatest sensitivity to contextual criteria. Even so, only 17 interactions are reclassified in its output when changing from CA1 to CA4 across S_{A2}-S_{B*2} corpora, compared to 164 for REACH and 154 for Llama 3.

8 Database-supported reader output retention

Figure S7 provides retention values for each subcategory and each reader across corpora. Summarized across R_{A2}-R_{B*1}, LLM output has higher retention rate than NLP in most subcategories (Figure S7A). The outliers with 100% retention are cases where only one interaction was extracted in the subcategory and retained, and therefore, using the largest corpus is a better indicator of the quality of reader output. For R_{A1}, REACH and INDRA have similar retention rate in most subcategories (Figure S7B). Consistently for all corpora, INDRA did not provide strong corroborations and attribute conflicts due to its lower attribute coverage.

Internal extensions are more likely to be supported by interaction databases, as they are the only extension subcategory where interactions match both model elements. This is confirmed by results (Figure S7A,B), which show that internal extensions are filtered out significantly less than full and hanging extensions.

We note that the number of interactions considered within classification subcategories can vary several orders of magnitude (Figure S7A,B - line), and only ~10 total strong corroborations are retained across all corpora, versus ~10,000 retained extensions for R_{A1}. Lower than expected retention of strong corroborations is likely due to the filtering tool removing all protein family names (e.g., AKT) and keeping only specific proteins (e.g., AKT1, AKT2), which results in filtering out even some interactions that VIOLIN classified as strong corroborations. Thus, VIOLIN may be less restrictive in some cases, considering a match as long as the same element ID and element type are used.

9 Benchmark details and evaluation results

We created several groups of manually curated benchmark interaction lists. All interaction lists are in the form of Excel files and included in the VIOLIN GitHub repository (https://github.com/pitt-miskov-

zivanov-lab/VIOLIN/tree/master/eval). The summary of lists is provided in Figure S8A. All scripts and the spreadsheets with results are also available on GitHub.

Group 1 has 14 fully populated interactions, with one interaction in each subcategory with respect to model A. These interactions were created manually to test the correctness of the algorithm implementation - whether VIOLIN follows the decision tree outlined in Figure 2A.

Group 2 includes two interactions lists with interactions from either model A (266 interactions) or model B (70 interactions). These two benchmark lists were created with a script that converts models to corresponding interaction lists and are used to test the accuracy of classifying into the corroboration category. The translator is available as part of the UI, under BioRECIPE (https://nmzlab.github.io/Tools-UI), as well as a separate script code on GitHub (https://github.com/pitt-miskov-zivanov-lab/BioRECIPE/tree/main/translators/within_biorecipe).

Group 3 includes two interaction lists that were created by adding 10 contradictions for models A and B to the two corresponding lists in Group 2.

Group 4 includes two interaction lists that were created by adding 10 randomly selected and manually classified interactions from REACH and INDRA outputs to interaction lists in Group 2.

Group 5 was created to evaluate the effectiveness and human expert alignment of the algorithm on a larger set. Since manual curation of interaction lists is slow, we chose two interaction lists from REACH output, one from each set A and B, R_{A2} and R_{B2} (sizes listed in Figure S4). For these two lists, we created new sub-lists M_0, M_1, M_2, and M_3 (Figure S8B). The two M_0 lists were created by removing interactions where the regulator or regulated element name either contains multiple entities, begins with a number, or consists solely of special characters (e.g., #, /, ~, $, etc.). R_{A2}-M_0 list has 5,274 unique interactions, R_{B2}-M_0 list has 411 unique interactions. M_1 was formed from M_0 by keeping only the interactions that do not have any element matches with the model. M_2 consists of interactions where exactly one element matches a model element. The remaining interactions that match the model in both elements formed list M_3. By construction, all interactions in lists M_1 are full extensions and all interactions in lists M_2 are hanging extensions, with respect to their corresponding model. Interactions in M_3 lists are either corroborations, contradictions, flagged, or internal extensions, with respect to their corresponding model. The two M_3 lists were manually classified to serve as a benchmark for evaluating VIOLIN's classification.

Group 6 includes only those interactions from Group 5 that were retained after filtering with FLUTE, reducing lists R_{A2}-M_0 and R_{B2}-M_0 to 870 and 37 interactions, respectively (Figure S8C).

As expected, when compared to manual curation, VIOLIN had 100% correct performance on all benchmarks in Groups 1-4 (Figure S8A). In Group 5, VIOLIN has 100% precision and recall for benchmark M_1 in R_{B2} and has >97.5% recall for M_1 in R_{A2} (3 out of 2,250 interactions misclassified). Inspection of misclassified interactions confirms again that VIOLIN correctly implements the decision tree in Figure 2A and that the errors stem from the REACH entity recognition and grounding – while an expert is able to recognize nuances in element and interaction attributes, VIOLIN's string processing was not created to handle errors and assumes correctly grounded entities. In our future work, we will explore methods to improve name recognition in VIOLIN, possibly using LLMs. To confirm that VIOLIN can indeed correctly classify interactions that are properly grounded, using Group 6 benchmarks we examined classification accuracy of the interactions that were retained after filtering with FLUTE. Since these interactions are highly supported by databases, they are less likely to have grounding errors, and therefore, should be accurately classified by VIOLIN.

Notably, upon initial inspection of misclassified interactions, we found that VIOLIN corrected the human curator in several instances – VIOLIN was able to identify a matching interaction or pathway in the model that the human curator missed. These results highlight VIOLIN's ability to both correct human errors and significantly accelerate the curation process beyond what is possible with manual methods.

10 Extended runtime and complexity analysis

The total VIOLIN runtime T_T is calculated as follows:

$$T_T = T_L + T_C + T_W$$

T_L – loading time – the time necessary to read the baseline model file (or interaction list) and the new interaction list file

T_C – classification time – the time necessary to process an interaction list with VIOLIN

T_W – write time – the time necessary to create output files for the four categories

We also computed the average classification speed as:

$$s_C^{avg} = avg(s_{C,REACH}, s_{C,INDRA}, s_{C,ChatGPT3.5}, s_{C,Llama2})$$

where the classification speed for each reader output is calculated as a ratio between the number of interactions that VIOLIN treats as unique in the reader output and the classification time for those interactions:

$$s_{c,reader} = \frac{N_{VIOLIN\ unique,reader}}{T_{C,reader}}$$

The average total processing speed is calculated as:

$$s_T^{avg} = avg(s_{T,REACH}, s_{T,INDRA}, s_{T,ChatGPT3.5}, s_{T,Llama2})$$

where the total processing speed for each reader output is calculated as a ratio between the total number of interactions output by the reader and the total time needed for VIOLIN to load input files, classify interactions, and write output files for that reader output:

$$s_{T,reader} = \frac{N_{T,reader}}{T_{T,reader}}$$

Algorithm complexity analysis. VIOLIN takes two inputs: an interaction list containing n interactions and a model whose graph topology consists of m nodes and e edges. VIOLIN processes one interaction per iteration, where finding matching nodes in the baseline graph is approximately $O(m)$. In the worst case, a search for a path between matched nodes is necessary, and VIOLIN then invokes a path-finding Dijkstra's algorithm, contributing $O((m + e) \cdot \log m)$ per iteration. Therefore, the total time complexity is bounded between $O(n \cdot m)$ and $O(n(m + (m + e) \cdot \log m))$. We also note that string matching for entities may vary due to different algorithms, and here we treat it as $O(1)$ and exclude it from this analysis.

References

1. Holtzapple, E., Luo, H., Tang, D., Zhou, G., Arazkhani, N., Hansen, C., Telmer, C.A., and Miskov-Zivanov, N. (2024). The BioRECIPE Knowledge Representation Format. bioRxiv, 2024.2002. 2012.579694.

2. BioRECIPE: Biological system Representation for Evaluation, Curation, Interoperability, Preserving, and Execution. (2023). https://melody-biorecipe.readthedocs.io/en/latest/index.html.

3. BioRECIPE GitHub Repository. (2024). https://github.com/pitt-miskov-zivanov-lab/BioRECIPE/tree/main.

4. VIOLIN GitHub Repository. (2024). https://github.com/pitt-miskov-zivanov-lab/VIOLIN.

5. Ashburner, M., Ball, C.A., Blake, J.A., Botstein, D., Butler, H., Cherry, J.M., Davis, A.P., Dolinski, K., Dwight, S.S., Eppig, J.T., et al. (2000). Gene Ontology: tool for the unification of biology. Nature Genetics *25*, 25-29. 10.1038/75556.

6. Consortium, T.U. (2018). UniProt: a worldwide hub of protein knowledge. Nucleic Acids Research *47*, D506-D515. 10.1093/nar/gky1049.

7. PubMed Central. (2005). Reference Reviews *19*, 37-38. doi:10.1108/09504120510587797.

8. Valenzuela-Escárcega, M.A., Hahn-Powell, G., Surdeanu, M., and Hicks, T. (2015). A Domain-independent Rule-based Framework for Event Extraction. held in Stroudsburg, PA, USA, (Association for Computational Linguistics and The Asian Federation of Natural Language Processing), pp. 127-132.

9. Akbani, R., Akdemir, Kadir C., Aksoy, B.A., Albert, M., Ally, A., Amin, Samirkumar B., Arachchi, H., Arora, A., Auman, J.T., Ayala, B., et al. (2015). Genomic Classification of Cutaneous Melanoma. Cell *161*, 1681-1696. https://doi.org/10.1016/j.cell.2015.05.044.

10. Miskov-Zivanov, N., Turner, M.S., Kane, L.P., Morel, P.A., and Faeder, J.R. (2013). The duration of T cell stimulation is a critical determinant of cell fate and plasticity. Sci Signal *6*, ra97. 10.1126/scisignal.2004217.

Table S1. Five examples of interaction attributes used by VIOLIN's decision algorithm.

		Interaction attributes		Interaction attribute examples				
		Attribute name	Attribute symbol	1	2	3	4	5
Elements (nodes, v)	Regulator (source node, v_s)	Name*	a_s^{name}	CK1	mTOR	Akt	Resveratrol	RAS
		Type*	a_s^{type}	protein	protein	protein family	chemical	protein family
		Subtype	$a_s^{subtype}$	kinase	kinase	kinase	antioxidant	GTPase
		HGNC Symbol*	$a_s^{HGNCsymbol}$	CSNK1A1	MTOR	AKT1,AKT2, AKT3	N/A	HRAS, KRAS, NRAS
		Database*	$a_s^{database}$	UniProt	UniProt	FamPlex	CHEBI	PFAM
		ID*	a_s^{ID}	P48729	P42345	AKT	445154	PF00071
		Compartment	$a_s^{compartment}$	cytoplasm	cytoplasm, nucleus	cytoplasm, membrane	N/A	other
		Compartment ID	$a_s^{compartmentID}$	GO: 0005737	GO:0005737, GO:0005634	GO:0005737, GO:0016020	N/A	GO:0016020
	Regulated (target node, v_t)	Name*	a_t^{name}	APC	Chk1	GSK3beta	PTEN	p110gamma
		Type*	a_t^{type}	protein	protein	protein	protein	protein
		Subtype	$a_t^{subtype}$	tumor suppressor	kinase	kinase	phosphatase	enzyme
		HGNC Symbol*	$a_t^{HGNCsymbol}$	APC	CHEK1	GSK3B	PTEN	PIK3CG
		Database*	$a_t^{database}$	UniProt	UniProt	UniProt	UniProt	UniProt
		ID*	a_t^{ID}	P25054	O14757	P49841	P60484	P48736
		Compartment	$a_t^{location}$	cytoplasm	cytoplasm, centrosome	cytoplasm, nucleus, membrane	cytoplasm, nucleus	other
		Compartment ID	$a_t^{locationID}$	GO: 0005737	GO: 0005737, GO:0005813	GO:0005737, GO:0005634, GO:0016020	GO:0005737, GO:0005634	GO:0016020
Interaction (edge, e)	Influence	Sign*	a_e^{sign}	positive	positive	negative	positive	positive
		Connection Type	$a_e^{connectiontype}$	direct	indirect	indirect	indirect	indirect
		Mechanism	$a_e^{mechanism}$	phosphorylation	amount	phosphorylation	transcription	activation
		Site	a_e^{site}	S1504, S150, S1507, S1510	N/A	S9	N/A	N/A
	Context	Cell Line	$a_e^{cellline}$	SW480	cellosaurus:CVCL_0045	CHO	LNCaP, DU145	COS-7
		Cell Type	$a_e^{celltype}$	colorectal cancer	embryonic	epithelial	prostate cancer	fibroblast-like
		Tissue Type	$a_e^{tissuetype}$	large intestine	kidney	ovarian	brain, lymph node	kidney
		Organism	$a_e^{organism}$	human	human	human, mouse, hamster	human	human, monkey
	Proven.	Score	a_e^{score}	INDRA: 0.999	STRING: 0.763, INDRA:0.982	STRING: 0.996, INDRA:0.999	STITCH: 0.963, INDRA:0.999	INDRA:0.997
		Source	a_e^{source}	literature, expert, database	literature, expert, database	literature, expert, database	literature, expert, database	literature, expert, database
		Statements	$a_e^{statements}$	Sentence 1	Sentence 2	Sentence 3	Sentence 4	Sentence 5
		Paper IDs	$a_e^{paperIDs}$	PMC2654145, PMID11487578	PMC4381605	PMC1403772, PMC3535741	PMC3181262, PMC2957324	PMC2652403

Sentence examples:
1. "This may be analogous to parallel mechanisms that promote GSK3 phosphorylation of beta-catenin in the absence of Wnt stimulation, such as by GSK3 and CK1 phosphorylation of Axin and APC."
2. "mTOR inhibition in HEK293 cells significantly reduced the total Chk1 level"
3. "Therefore, given that Akt phosphorylates and inactivates GSK3beta, we hypothesized that Akt dependent inactivation of GSK3beta might be responsible for Notch potentiation."
4. "Our results demonstrate that resveratrol induced the expression of PTEN…"
5. "Ras activates p110gamma at the level of the membrane, by allosteric modulation and/or reorientation of the p110gamma…"

Attributes marked with an asterisk (*) are included in the required match conditions.

Sentences from which interaction examples were extracted:

1. "This may be analogous to parallel mechanisms that promote GSK3 phosphorylation of beta-catenin in the absence of Wnt stimulation, such as by GSK3 and CK1 phosphorylation of Axin and APC."
2. "mTOR inhibition in HEK293 cells significantly reduced the total Chk1 level"
3. "Therefore, given that Akt phosphorylates and inactivates GSK3beta, we hypothesized that Akt dependent inactivation of GSK3beta might be responsible for Notch potentiation."
4. "Our results demonstrate that resveratrol induced the expression of PTEN…"
5. "Ras activates p110gamma at the level of the membrane, by allosteric modulation and/or reorientation of the p110gamma…"

Table S2. Summary of paper retrieval approaches, reader output, interaction list sizes, and VIOLIN runtime for each paper corpus and corresponding interaction list for CS1, CA1.

Paper set	Paper retrieval approach	Query	Total papers	Reader	Interaction list	Total interactions	Unique interactions	Unique VIOLIN interactions	Time (s)	Average time (s)
S_{A1}	PubMed search	melanoma AND (p70S6K OR S6 OR gsk OR gsk3 OR gsk3a OR gsk3b OR src OR 4ebp1 OR "eIF4E-binding protein 1" OR PHASI OR ybi OR YBX1 OR NSEP1 OR YB1 OR CRD OR Y-box OR SkMel-133)	6039	REACH	R_{A1}	131445	105186	125545	402.254	3.06E-03
				INDRA		72531	49837	51628	73.3973	1.01E-03
				GPT-4.1		-	-	-	-	-
				Llama 3		-	-	-	-	-
S_{A2}	REACH Explorer, Fetch	MEK, ERK, AKT, GSK3, P70RSK, S6, CDK4, 4EBP1, YB1, SRC, CHK2, MTOR, PI3K	120	REACH	R_{A2}	6305	5200	5907	3.9387	6.25E-04
				INDRA		1848	1454	1524	0.8625	4.67E-04
				GPT-4.1		2736	2640	2734	1.8008	5.91E-04
				Llama 3		5198	4920	5177	3.9539	5.65E-04
S_{A3}	REACH Explorer	MAPK/ERK pathway	50	REACH	R_{A3}	1471	1352	1437	0.7782	5.29E-04
				INDRA		593	542	559	0.3053	5.15E-04
				GPT-4.1		1272	1255	1272	0.7994	5.33E-04
				Llama 3		1924	1853	1917	1.3654	5.86E-04
S_{A4}	REACH Explorer	RPS6K1	3	REACH	R_{A4}	59	51	54	0.04578	7.76E-04
				INDRA		30	30	30	0.03521	1.17E-03
				GPT-4.1		70	70	70	0.0709	1.08E-03
				Llama 3		111	111	111	0.1215	2.01E-03
S_{B1}	REACH Explorer	PTEN	43	REACH	R_{B1}	1168	944	1124	0.5304	4.54E-04
				INDRA		734	579	619	0.3065	4.18E-04
				GPT-4.1		816	777	815	0.4603	4.74E-04
				Llama 3		986	771	965	0.6159	5.57E-04
S_{B2}	Hawse et al. references	N/A	13	REACH	R_{B2}	440	403	432	0.2111	4.80E-04
				INDRA		380	351	363	0.3065	8.07E-04
				GPT-4.1		249	245	248	0.1516	4.90E-04
				Llama 3		405	382	404	0.2781	7.75E-04
S_{B3}	REACH Explorer	T-cell, PTEN, AKT, FOXO	11	REACH	R_{B3}	373	353	369	0.1834	4.92E-04
				INDRA		389	374	384	0.181	4.65E-04
				GPT-4.1		304	299	304	0.1889	5.44E-04
				Llama 3		367	332	363	0.2396	7.75E-04
S_{B*1}	REACH Explorer	Breast Cancer, DNA repair, Autophagy, Cancer	38	REACH	R_{B*1}	1223	1104	1124	0.5304	4.54E-04
				INDRA		847	759	619	0.3065	4.18E-04
				GPT-4.1		837	814	836	0.4603	4.74E-04
				Llama 3		1111	1038	1099	0.6159	5.57E-04
S_{B*2}	REACH Explorer	DNA repair, BRCA1, ADAM17, inflammation	15	REACH	R_{B*2}	871	807	432	0.2111	4.80E-04
				INDRA		634	573	363	0.3065	8.07E-04
				GPT-4.1		274	270	272	0.1516	4.90E-04
				Llama 3		483	458	479	0.2781	7.75E-04

Total interactions – interactions retrieved or extracted from database or literature

Unique interactions – set of interactions obtained by removing duplicates (interactions with the same: regulator name/id/type, regulated name/id/type, interaction sign) from Total interactions

Unique VIOLIN interactions – set of interactions obtained by removing duplicates from Total interactions by VIOLIN (VIOLIN duplicates are interactions that have same elements, context, and influence attributes)

Time – time of executing the VIOLIN code with the new interaction list and baseline model files as inputs (T_T).

Average time – average time per interaction ($^1/_{s_{T,reader}}$)

A.

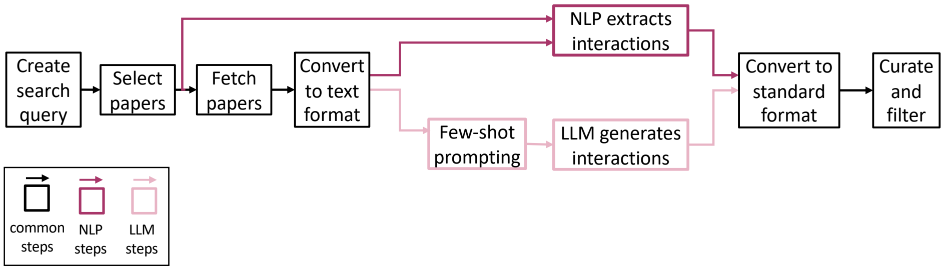

B.

Legend: -- advantage ✓ – present/required; -- drawback x – absent/not req.

Tool	Approach	Available features: PubMed access	Paper search	Database access	Open access	API access	Add. steps and resources: Pre-processing	Prompting	Scripts	Parallelization	Large memory	Runtime: [min/paper]	Device
**REACH**	RB+Stat.	✓	✓	✓	✓	✓	x	x	x	x	x	~2	CPU
**INDRA**	RB+Stat.+ KB	✓	✓	✓	✓	✓	x	x	x	x	x	~0.5	
**GPT-4.1**	LLM-few shots	x	x	x	x	✓	✓	✓	✓	✓	✓	~8	API call
**Llama 3**	LLM-few shots	x	x	x	✓	✓	✓	✓	✓	✓	✓	~1.9	GPU

C.

You are an expert biomedical assistant. Your task is to:
extract relevant information from biomedical research papers and strictly follow the example below,
extract the biological intracellular interaction lists (Biological Regulator, Biological Regulated, Biological Interaction) from the given text.
for Interaction Sign choices: {SIGN_CHOICES},
for Interaction Connection Type choices: {CNX_TYPE_CHOICES},
for Biological Regulator and Biological Regulated Type choices: {TYPE_CHOICES},
for Biological Regulator and Biological Regulated Database choices: {DATABASE_CHOICES},
for Biological Regulator and Biological Regulated Compartment choices: {COMPARTMENT_CHOICES},
for Interaction Mechanism choices: {MECHANISM_CHOICES}.

Q: B-Raf phosphorylates MEK1 and MEK2 on Ser217 and Ser221 , which activates it to dual phosphorylate ERKs , at Thr202 and Tyr204 for human ERK1 and Thr185 and Tyr187 for human ERK2 [XREF_BIBR , XREF_BIBR] .

A:
{INTERACTION_1}
{INTERACTION_2}
{INTERACTION_3}
{INTERACTION_4}

Q: Inhibition of NF-kappaB by Gliotoxin , MG132 or Sulfasalazine [XREF_BIBR] sensitizes pancreatic cancer cells to apoptosis induced by etoposide (VP16) or doxorubicin .

A:
{INTERACTION_1}
{INTERACTION_2}

Q: {text}

SIGN_CHOICES	[positive, negative]
CNX_TYPE_CHOICES	[direct, indirect]
TYPE_CHOICES	[gene, protein, RNA, chemical, protein family, biological process]
DATABASE_CHOICES	[UniProt, HGNC, PubChem, Ensembl, GENCODE ,RefSeq ,Pfam, InterPro, GO, MeSH]
COMPARTMENT_CHOICES	[cytoplasm, cytosol, plasma membrane, nucleus, mitochondria ,endoplasmic reticulum ,extracellular]
MECHANISM_CHOICES	[phosphorylation, dephosphorylation, ubiquitination, deubiquitination, acetylation, deacetylation, methylation, demethylation, transcription, translation, translocation]

INTERACTION_1	Biological Regulator:<br>Name: B-Raf<br>Type: protein<br>Subtype: Kinase<br>HGNC Symbol: BRAF<br>Database: uniprot<br>ID: P15056<br>Compartment: N/A<br>Compartment ID: N/A<br><br>Biological Regulated:<br>Name: MEK1<br>Type: protein<br>Subtype: Kinase<br>HGNC Symbol: MAP2K1<br>(Continue to right)	(cont'd)<br>Database: uniport<br>ID: Q02750<br>Compartment: N/A<br>Compartment ID: N/A<br><br>Interaction:<br>Sign: positive<br>Connection Type: direct<br>Mechanism: phosphorylation<br>Site: Ser217/Ser221<br>Cell Line: N/A<br>Cell Type: N/A<br>Tissue Type: N/A<br>Organism: N/A
INTERACTION_2	...	
...	...	

D.

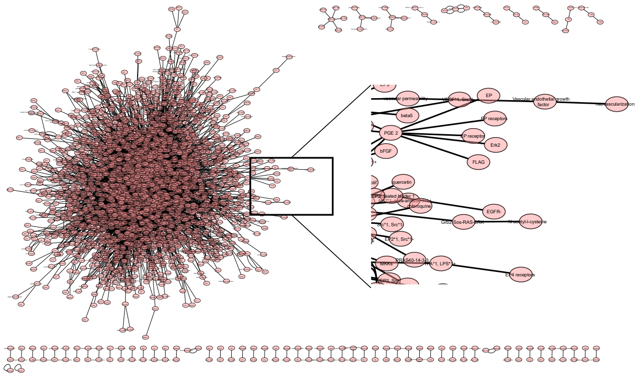

Figure S3. Overview of paper retrieval methods.

A. The workflow including steps for running rule-based NLP tools and prompting LLMs.

B. Summary of the approaches used, advantages and drawbacks of the four reader tools (RB – rule-based, Stat. – statistics, KB – knowledge base). Columns indicate Available features (advantages): programmatic/automated access to PubMed, paper retrieval, and interaction and pathway databases, open-access reader tool, and programmatic (API) access to the tool itself; Additional steps or resources that are required by the tool (disadvantages): paper preprocessing or reformatting before extracting interactions, prompting in order to "train" for suitable output format, additional scripts or code are necessary for running, parallelization for timely output, large memory necessary for processing. Average time per processed paper and the device on which the tool was run.

C. Prompt used with ChatGPT-4.1 and Llama3. Prompt comprises a system message specifying the available choices for each attribute and a list of extraction demonstrations, each consisting of a passage paired with a set of annotations. The table on the right presents allowed values for each variable in the prompt.

D. Network of interactions in the interaction list R_{A2}, which has 2200 nodes and 6305 edges, extracted by REACH for corpus S_{A2}.

A. B.

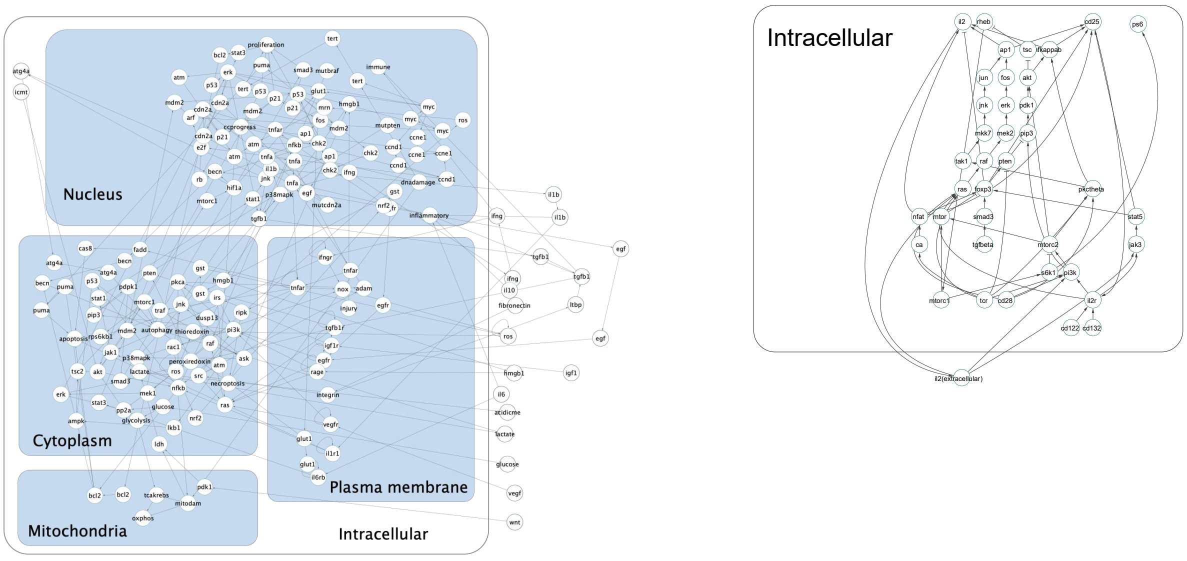

C.

#	Baseline model	Biological context	Number of nodes	Number of edges	Corresponding corpora
1	**A**	Melanoma cell line SkMel-133	179	266	S_{A1}, S_{A2}, S_{A3}, S_{A4}
2	**B**	Naïve T-cell differentiation to Treg and Th	39	70	S_{B1}, S_{B2}, S_{B3}, S_{B*1}, S_{B*2}
3	**PID: 8615**	IL23-mediated signaling events	78	38	S_{A2}
4	**PID: 11552**	IL27-mediated signaling events	63	26	S_{A2}
5	**PID: 22080**	ErbB1 downstream signaling	287	46	S_{A2}
6	**PID: 52883**	IL5-medicated signaling events	56	4	S_{A2}
7	**PID: 61956**	PDGFR-beta signaling pathway	374	122	S_{A2}
8	**PID: 72464**	IFN-gamma pathway	116	29	S_{A2}
9	**PID: 73660**	IL6-mediated signaling events	113	39	S_{A2}
10	**PID: 81472**	IL12-mediated signaling event	118	36	S_{A2}
11	**Reactome: R-HAS-69231**	Cyclin D associated events in G1	198	25	S_{A2}
12	**Reactome: R-HAS-982772**	Growth hormone receptor signaling	105	21	S_{A2}
13	**Reactome: R-HAS-1059683**	Interleukin-6-signaling	70	8	S_{A2}
14	**Reactome: R-HAS-2559582**	Senescence-Associated Secretory Phenotype (SASP)	157	27	S_{A2}
14	**Reactome: R-HAS-2586552**	Signal by Leptin	52	12	S_{A2}
16	**Reactome: R-HAS-6785807**	Interleukin-4 and Interleukin-13 signaling	303	29	S_{A2}
17	**Reactome: R-HAS-9020956**	Interleukin-27 signaling	51	10	S_{A2}

Figure S4. Networks of baseline graphs A and B.

A. Network of the melanoma cell line SkMel-133, used as baseline graph A.

B. Network of the circuitry that controls T cell differentiation we use as a baseline model B with 39 nodes and 70 edges.

C. Summary of baseline graphs used, their sizes and corresponding literature corpora. Table lists the IDs and names of graphs that are acquired from external databases, Pathway Interaction Database (PID) and Reactome. Query used to acquire pathway networks through PathwayCommons: “melanoma, BRAF, MEK, ERK, IL6, STAT3, JAK2, CDK4, CDK6”.

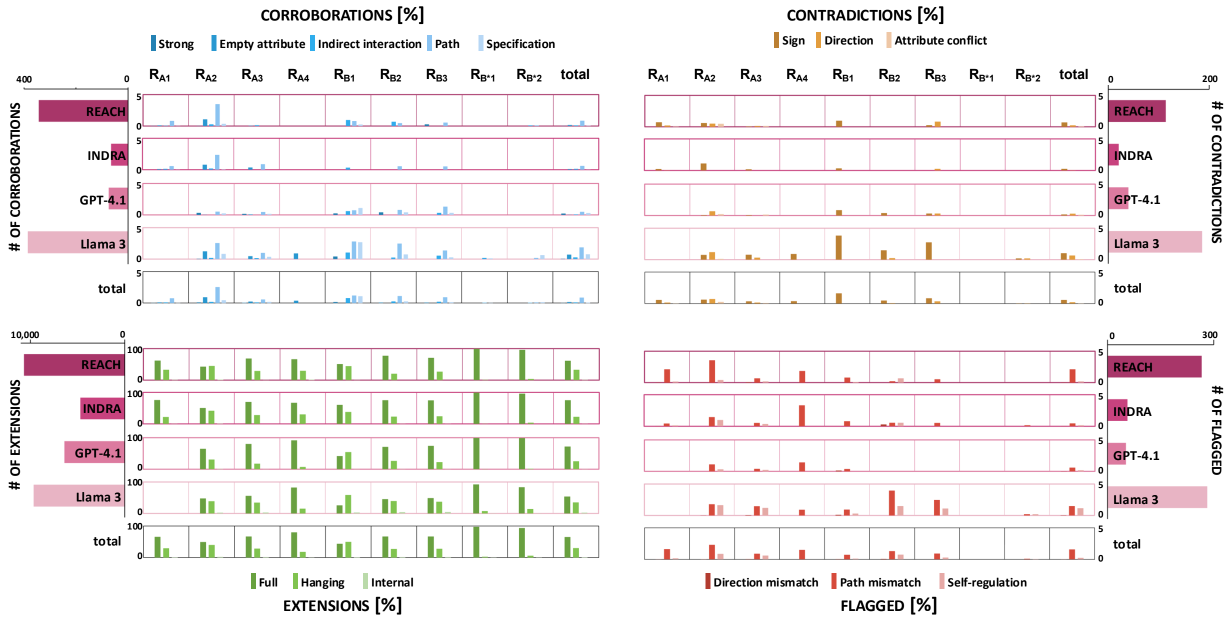

Figure S5. VIOLIN output for the four reader tools and all nine interaction lists.

Classification results for all nine interaction lists ($R_{A1\text{-}4}$, $R_{B1\text{-}3}$, and $R_{B*1\text{-}2}$), and all four readers (REACH, INDRA, GPT-4.1 and Llama 3), across all categories (Corroborations, Contradictions, Flagged, and Extensions) and subcategories.

Four category tables (middle): (columns – individual interaction lists) bar heights represent percentages out of total number of interactions in the list for each reader and across all readers; (columns – 'total') bar heights represent percentage for subcategories out of total number of interactions extracted across all lists by an individual reader; (rows – 'total') bar heights represent percentages for subcategories out of total number of interactions for each individual interaction list across all readers; (bottom right corner of each category table) bar heights represent percentages for subcategories out of total number of interactions across all lists and all readers.

Pink bar charts (left and right): the number of interactions extracted by each reader in each category (this excludes list R_{A1} for all readers, since GPT-4.1 and Llama3 were not used on this corpus)

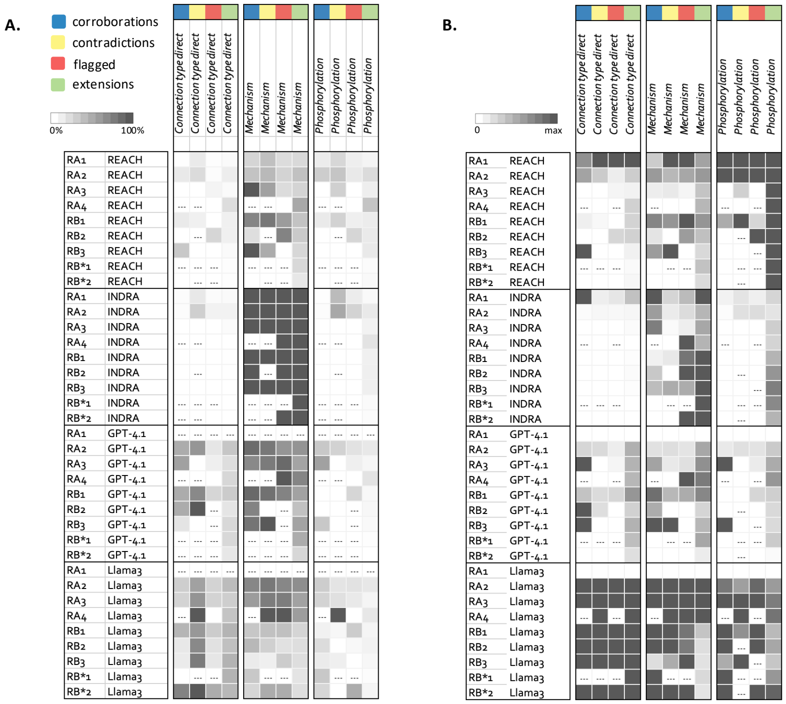

Figure S6. Attribute coverage for all readers across all corpora and classification categories.

A. Percent presence within extracted interaction list of "direct" in the Connection type attribute, any value for attribute Mechanism, and "phosphorylation" in the Mechanism attribute. Percents are listed relative to each corpus, for each reader.

B. Number of interactions with attribute value present for each corpus, each reader, and within each classification category. Maximum number is determined within each category and corpus across all readers. All results are obtained under scheme CS1 and strategy CA4.

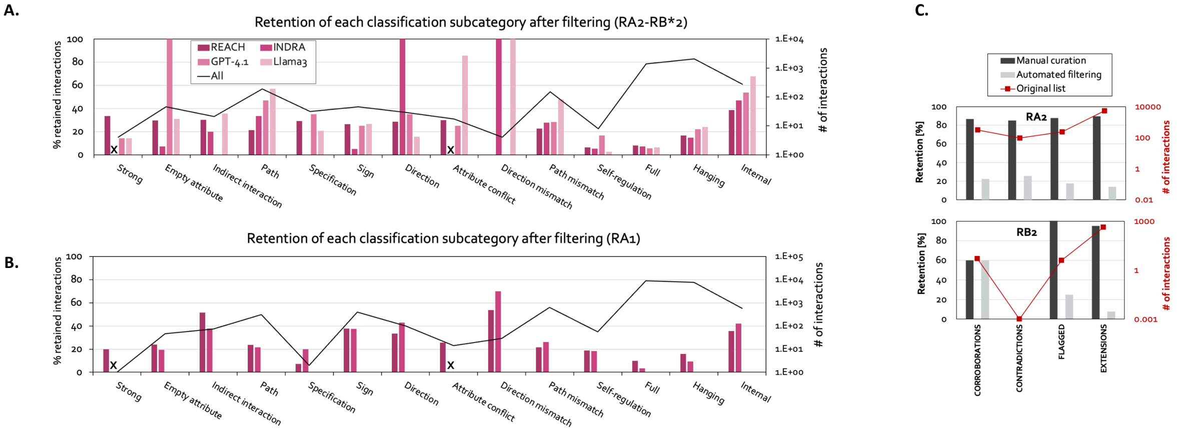

Figure S7. Retention of interactions after filtering and manual curation.

A. Bars- fraction (%) of interactions retained after filtering shown for each classification subcategory and each reader, computed across corpora R_{A2}-R_{B*2}. Line- total number of interactions retained after filtering in each subcategory summed across all readers and across corpora R_{A2}-R_{B*2}.

B. Bars- fraction (%) of interactions retained after filtering shown for each classification subcategory for REACH and INDRA and corpus RA1. Line- total number of interactions retained after filtering in each subcategory summed for REACH and INDRA for corpus R_{A1}. “x” indicates that there were no interactions in that subcategory before filtering.

C. Comparison of FLUTE filtering and manual curation for REACH output, lists R_{A2} and R_{B2}.

A.

Benchmark #	Benchmark Group	Interaction list Description	Interaction list Size	Precision	Recall	Accuracy	F1
1	1	One from each sub-category	14	1	1	1	1
2	2	Model A interactions	266	1	1	1	1
3		Model B interactions	70	1	1	1	1
4	3	A + 10 contradictions	276	1	1	1	1
5		B + 10 contradictions	80	1	1	1	1
6	4	A + 10 random interactions	276	1	1	1	1
7		B + 10 random interactions	80	1	1	1	1
8	5	R_{A2}-M_1	2250	0.998	0.998	0.999	0.998
9		R_{A2}-M_2	2425	0.976	0.976	0.988	0.976
10		R_{A2}-M_3	599	0.846	0.846	0.923	0.846
11		R_{B2}-M_1	309	1	1	1	1
12		R_{B2}-M_2	94	0.989	0.989	0.9995	0.989
13		R_{B2}-M_3	8	0.875	0.875	0.938	0.875
14	6	R_{A2}-M_1+FLUTE	222	1	1	1	1
15		R_{A2}-M_2+FLUTE	405	1	1	1	1
16		R_{A2}-M_3+FLUTE	171	0.977	0.977	0.988	0.977
17		R_{B2}-M_1+FLUTE	18	1	1	1	1
18		R_{B2}-M_2+FLUTE	15	1	1	1	1
19		R_{B2}-M_3+FLUTE	4	1	1	1	1

B.

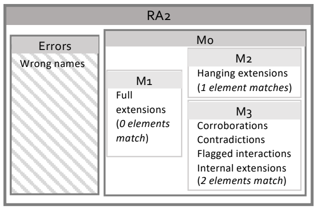

C.

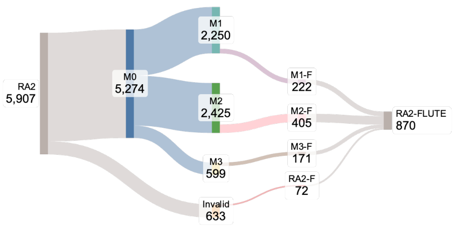

D.

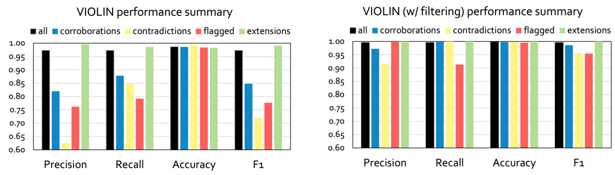

Figure S8. VIOLIN testing.

A. Summary of tests conducted on VIOLIN. All interactions lists can be found in VIOLIN's GitHub repository.

B. Illustration of how tests M_0-M_3 are organized (shown with RA2, same for RA3).

C. Sankey diagram showing interaction list sizes, starting from the original list R_{A2}, through list M_0, then lists M_1, M_2, M_3, and their corresponding sub-lists after filtering with FLUTE, M_1-F, M_2-F, M_3-F. "invalid" indicates interactions that were removed from R_{A2} with manual curation when creating list M_0. The numbers indicate total interaction list size obtained from REACH, before finding redundant interactions.

D. Performance summary across all corpora and all readers, for each category, without (left) and with (right) pre-filtering.
